# Impacts of Mesoscale Eddy Structural Characteristics on Matched-Field Localization Uncertainty

**DOI:** 10.3390/s25226842

**Published:** 2025-11-08

**Authors:** Longquan Shang, Kaifeng Han, Ning Wang, Yanqun Wu, Guojun Xu, Pingzheng Li, Wei Guo

**Affiliations:** 1 College of Meteorology and Oceanography, National University of Defense Technology, Changsha 410073, Chinaxuguojun@nudt.edu.cn (G.X.);; 2 National Key Laboratory of Underwater Acoustic Technology, Harbin Engineering University, Harbin 150001, China

**Keywords:** mesoscale eddy, vertical structure composite analysis, matching field processing, error distribution

## Abstract

**Highlights:**

**What are the main findings?**

- The distribution of localization errors caused by eddies exhibits regularity.
- The mechanism underlying the regularity of localization error distribution.

**What are the implications of the main findings?**

- The localization errors caused by eddies are limited.
- The parameters of eddies can theoretically be obtained through matched field inversion.

**Abstract:**

Matched-field processing localizes underwater acoustic targets by measuring the degree of correlation between the acoustic field and replica fields. The intrusion of mesoscale eddies can induce sound speed mismatch in the matched-field process. Therefore, it is essential to investigate the impact of mesoscale eddies on matched-field localization errors. In this study, the typical vertical structure of mesoscale eddies in a certain region of the Northwestern Pacific was synthesized using the mesoscale eddy dataset META 2.0 and Argo float data. Furthermore, by employing both an idealized eddy model and composite-analysis structure of eddy, the performance of the localization algorithm was evaluated under the influence of mesoscale eddies with different structures and in different regions. The results show that under specific conditions, the distribution of localization errors exhibits certain patterns, which is beneficial for inverting eddy parameters via matched-field processing. Finally, the mechanism behind the systematic distribution of localization errors is discussed and analyzed. In the simulations, the source frequency was swept from 50 to 75 Hz with a 1 Hz step, and a circular array was employed as the receiving aperture. These findings indicate that, in the absence of small-scale interference and within a certain range of sound speed mismatch, the localization error of underwater acoustic targets increases with the strengthening of mesoscale eddy disturbances.

## 1. Introduction

As a ubiquitous dynamic phenomenon in the ocean, mesoscale eddies significantly perturb the sound speed profile by altering the temperature-salinity structure and flow field distribution of seawater, thereby modulating the characteristics of underwater acoustic propagation [1,2]. An Experiment in the Sargasso Sea was the first to reveal the acoustic field perturbation effect of eddies [3]. Through simulations, Baer found that the acoustic energy received within a specific angular range by the receiver exhibits significant fluctuations after sound waves pass through an eddy [4]. Heaney investigated the horizontal refraction effect of eddies on sound waves [5]. Under certain conditions, mesoscale eddies can form surface ducts and subsurface ducts [6,7], enabling the long-distance propagation of sound waves [8,9]. The modulation effect of eddy on the interference pattern of the sound field was investigated [10]. Since the 1980s, a series of experiments have been carried out, such as the SLICE89 experiment, Acoustic Engineering Test, Alternate Source Test, ATOC experiment, PhiSea09 experiment, and OBSAPS experiment [11,12,13]. These experiments have systematically explored the mechanism by which marine environmental variability affects acoustic propagation and confirmed that eddy activity exerts observable modulation effects on acoustic propagation reality, modal structure, and group velocity. Additionally, a parameterization scheme for sound speed distribution is proposed [14].

Matched field processing is a widely used technology in underwater target localization and a key issue in the field of underwater acoustics. In 1973, Hinich first used a vertical array to verify sound source localization and derived the concept of the maximum likelihood estimator [15]. Bucker introduced the concept of the ambiguity surface and proved that the acoustic field possesses sufficient spatial complexity to enable acoustic field localization through inversion [16]. Klemm improved the linear processor and obtained a super-linear generalized maximum entropy beamformer, which further enhanced the localization accuracy in sound source depth estimation [17]. Subsequently, based on this, Capon proposed the currently most commonly used Minimum Variance Distortionless Response (MVDR) matched field processor, i.e., the MVDR beamformer [18]. The effectiveness of these theories and methods has been verified in real-world experiments. However, Environmental mismatches, especially in bottom bathymetry and array tilt, cause the matched field processing (MFP) peak to remain coherent but shifted in position, without significant degradation [19].

The localization accuracy of matched field processing technology is affected by various factors, such as noise, environmental mismatch, and system mismatch. Among these, environmental parameters, as system inputs, have a significant impact on the final localization results. The MVDR and many adaptive matched field algorithms are highly sensitive to environmental mismatch. Therefore, extensive research has been conducted to address the environmental mismatch problem, focusing on three main directions. Firstly, matched mode processing, which selects modes less affected by environmental parameters to participate in the matching process [20,21,22,23]. Secondly, environmental correction, such as using Bayesian models to estimate parameters [24]. Thirdly, utilizing signal processing methods to reduce localization errors [25].

As a common and ever-changing dynamic phenomenon in the ocean, mesoscale eddies continuously perturb the hydrological state, thereby affecting the acoustic field and inevitably exerting an impact on the results of matched field localization. In this study, the typical vertical structure of mesoscale eddies in a certain sea area of the Northwestern Pacific Ocean was synthesized. Using both the ideal eddy model and the synthetic structure model, the performance of localization algorithms under the influence of mesoscale eddies with different structures and in different regions was evaluated.

## 2. Materials and Methods

### 2.1. Gaussian Eddy Model

The Gaussian Eddy is an idealized eddy model commonly used in acoustics and oceanography. It is valued for its simple analytical form and smooth mathematical properties, making it particularly useful for studying the effects of eddies on acoustic wave propagation. From the perspective of acoustic studies, the distribution of the sound speed due to the existence of eddies can be parameterized by Gaussian eddy models [26,27]. It assumes that the spatial distribution of the sound speed consists of a linear superposition of the climatic state $c_{0}$ and the eddy-induced sound speed perturbations δc:

(1) $$c\left(r,z\right)=c_{0}\left(z\right)+\delta c\left(r,z\right)$$

**Figure 1:**
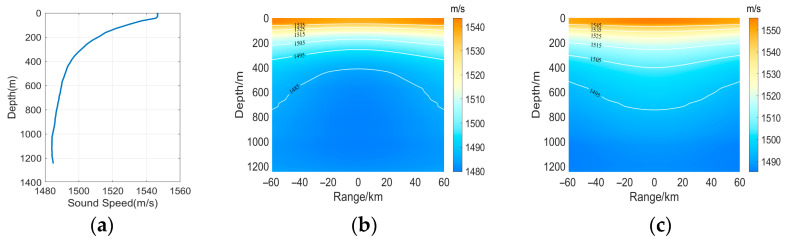
Sound speed profile. (**a**) no eddy, (**b**) cold eddy, (**c**) warm eddy.

The eddy-induced sound speed perturbation can be given by:

(2) $$\delta c\left(r,z\right)=Ae^{-{\left(\frac{r-L}{R}\right)}^{2}-{\left(\frac{z-D}{Z}\right)}^{2}}$$

where A is the intensity, indicating the maximum sound speed disturbance caused by the eddy. When A is negative, it corresponds to a cold eddy; when A is positive, it corresponds to a warm eddy. L denotes the horizontal coordinate of the eddy center. D denotes the vertical coordinate of the eddy center. A measured profile was selected as the background profile, as shown in Figure 1a. When $\left|A\right|$ = 10, the sound speed profile of the cold eddy is shown in Figure 1b, and that of the warm eddy is shown in Figure 1c.

### 2.2. Composite Analysis of the Vertical Structure of Mesoscale Eddies

Eddy-composite analysis refers to a methodology where: first, a large number of mesoscale eddies are identified using satellite sea surface height (SSH) data or eddy detection algorithms; second, the in situ profiles (from Argo floats, CTD profilers, drifting buoys, etc.) corresponding to the centers of these eddies are grouped based on eddy type, intensity, latitude, and other parameters; finally, temporal-spatial averaging is performed on the profiles of the same eddy category to derive an averaged structure representative of that eddy type [28,29]. The composite analysis of vertical structures of mesoscale eddies is based on statistical methods, which integrate a large amount of in situ observational data to reveal the typical structural characteristics of eddies. The synthesized structure reflects the common features of eddies, and through composite analysis, key characteristics such as common temperature and salinity anomalies in mesoscale eddies across different regions can be extracted. These features are physically interpretable and are often directly related to the dynamic mechanisms of eddies or associated currents. This technique is widely used in physical oceanography research [1,2,28,29,30,31,32]. On one hand, it helps address the scarcity of oceanographic survey data—individual eddy observations are limited, and satellite remote sensing can only capture sea surface information. The composite-analysis technique overcomes the limitations of single observation methods by integrating multi-source data (e.g., satellite altimetry data and Argo float data) to reconstruct three-dimensional structures. On the other hand, the synthesized structures of mesoscale eddies can provide benchmarks for numerical models and support the development of eddy parameterization schemes. The Northwestern Pacific Ocean is characterized by significant mesoscale eddy activity. This study synthesized the structures of mesoscale eddies using four sub-regions in this area, with the geographic coordinates of each sub-region detailed in Table 1. In Table 1, KE stands for the Kuroshio Extension, and STCC represents the Subtropical Countercurrent Region.

#### 2.2.1. Data

The Mesoscale Eddy Trajectory Atlas version 2.0 (META 2.0) is designed specifically for research on oceanic mesoscale eddies. Its raw data primarily originate from merged sea surface height measurements obtained by multiple satellite altimeters. Using an improved geometry-based eddy identification algorithm, eddies are automatically detected from the sea surface height field and tracked throughout their full lifecycle frame by frame [33,34]. The dataset covers sea surface characteristics of global mesoscale eddies from January 1993 to December 2019, including parameters such as amplitude, type, center coordinates, sequence number, maximum mean tangential velocity, radius, observation date, and trajectory ID. META 2.0 provides comprehensive sea surface dynamical information for integrated analysis of subsurface eddy structures and serves as a fundamental resource for studying the generation, propagation, decay, and energy cascade processes of mesoscale eddies.

The Argo Program (http://www.coriolis.eu.org, 10 August 2023) is a global ocean observation initiative centered around an array of thousands of autonomous profiling floats that conduct continuous and automated measurements of temperature and salinity from the sea surface down to a depth of 2000 m in the open global ocean [35]. These data are transmitted via satellite in near real-time. The dataset used in this study is the Argo float profile data released by the Coriolis Center, which has undergone automated quality control and processing. It includes underwater observations from 3 May 1998 to 11 January 2021, covering key variables such as pressure, temperature, salinity, float identifier, and coordinates. The high-accuracy temperature and salinity data from Argo are essential for synthesizing mesoscale eddies and revealing their vertical structure and dynamic mechanisms [30,31,32].

#### 2.2.2. Data Processing Procedure

Although the Argo data used had undergone automated quality control, visual inspection of the temperature and salinity profiles revealed issues such as missing variables, insufficient number of sampling points, and significant deviations from climatological means. To ensure the validity of subsequent analyses, further quality control was applied to the Argo profiles, which included three main steps: First, profiles with missing salinity or temperature data were excluded. This was necessary because some Argo observations prior to 2000 lacked either salinity or temperature records, preventing the calculation of sound speed profiles. Additionally, profiles containing null values (NaN) in temperature or salinity data were also removed. Second, profiles with fewer than 10 sampling points or those not reaching a depth of 1000 m were filtered out. Insufficient sampling points could lead to unrealistic vertical interpolation of temperature and salinity, compromising data reliability. Moreover, as mesoscale eddies typically influence depths shallower than 1000 m, profiles needed to cover at least this depth. Third, outliers were eliminated based on a threshold of three standard deviations. After matching Argo profiles to eddies and interpolating the data to standardized depths (10:1:1000 m), the mean and standard deviation of temperature and salinity at each depth were calculated across all profiles. Any profile deviating from the mean by more than three standard deviations at any depth was discarded.

**Figure 2:**
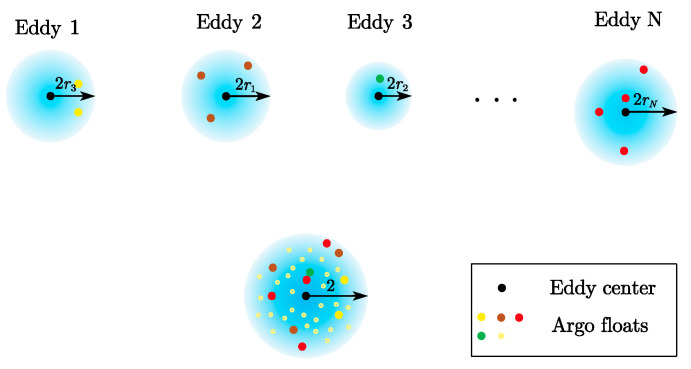
Schematic of the composite analysis of eddies. The black dots denote the geometric center of eddies. The colorful dots denote the Argo floats located within the circle area jointly determined by the eddy center and twice its radius. Here, the radius of the eddy ($r_{n},n=1,2,\cdot \cdot \cdot ,N$) is the radius of a circle whose area is equal to that enclosed by the contour of maximum circum-average speed.

The core methodology involved identifying all Argo profiles within the study area and matching each profile to the spatiotemporally closest eddy center. The matching criteria required the observation time to be identical and the Argo profile to be located within twice the radius of the eddy. The normalized distance of the float within the eddy coordinate system was calculated as the ratio of the distance between the float and the eddy center ($d_{i}$) to the eddy radius ($R_{i}$), denoted as $r_{i}=d_{i}/R_{i}$. The float was then projected into the eddy coordinate system (Figure 2). The synthesized temperature and salinity profile data from the eddy records were used to represent the ocean’s thermal and saline distribution [29,30,31,32,33]. Argo projections were completed, climatological temperature and salinity data were subtracted to obtain the typical structure of the eddy [32].

### 2.3. Conventional Matched Field Processing

Matched Field Processing (MFP) is a signal processing technique based on physical modeling of acoustic wave propagation, primarily used for underwater source localization and inversion of ocean environmental parameters. Its core principle is to compare the measured acoustic field with a theoretical replica field model to estimate the source location or properties of the marine environment. MFP is widely applied in sonar detection, ocean acoustic tomography, and related fields. With continued advances in MFP technology and improved understanding of underwater acoustic channel modeling, a variety of matched field processors have been developed. The most classical one is the Bartlett processor, which is essentially a spatial matched filter that computes the cross-correlation between the replica field vector and the actual data vector.

(3) $$B\left(r,z,f_{j}\right)=\frac{\left|\sum_{i=1}^{N}p_{i}^{e}\left(f_{j}\right)*p_{i}^{c}\left(f_{j}\right)\right|}{\sqrt{\sum_{i=1}^{N}{\left|p_{i}^{e}\left(f_{j}\right)\right|}^{2}\sum_{i=1}^{N}{\left|p_{i}^{c}\left(f_{j}\right)\right|}^{2}}}$$

$p_{i}^{e}\left(f_{j}\right)$ denotes the complex pressure signal spectrum received by the i-th hydrophone, while $p_{i}^{c}\left(f_{j}\right)$ represents the theoretically calculated complex sound pressure field which is computed using the coupled normal mode model in this paper [36]. Here, N is the number of hydrophones. By summing and averaging B from the previous formula across M frequency points, the inter-frequency non-coherent wideband Bartlett matched-field ambiguity surface function is obtained.

(4) $$\bar{B}=\frac{1}{M}\sum_{j=1}^{M}B\left(r,z,f_{j}\right)$$

## 3. Results

Mesoscale eddies do not exist in an idealized, homogeneous ocean. Their three-dimensional structure is significantly modulated by geographical conditions and surrounding current systems. For example, in the Kuroshio region, the water mass properties of eddies are profoundly influenced by major currents such as the Kuroshio and Oyashio [37], while the topography also varies considerably. The distinct oceanic structures in different regions exert varying impacts on the temperature and salinity fields, thereby inducing differential sound speed perturbations. These differences may subsequently lead to divergent localization errors in underwater acoustic positioning.

### 3.1. Anticyclone Eddy

**Figure 3:**
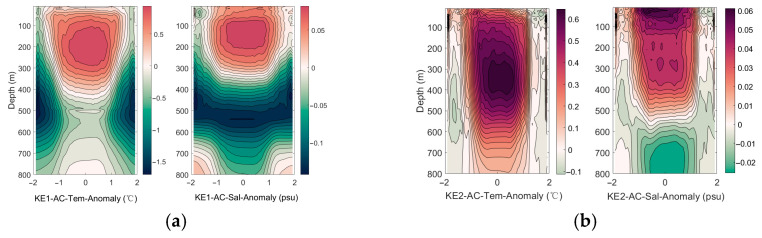

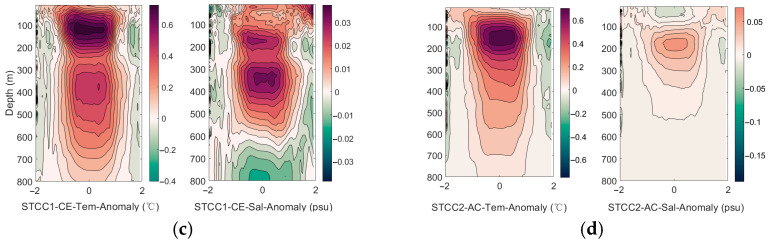
The horizontal axis represents the normalized radius, indicating the relative distance from the center of the anticyclone eddy (normalizing the radius of the eddy to 1). The range of −2 to 2 indicates the distance extending approximately twice the radius of the eddy outward from the center of the eddy. The coloring variable is temperature anomaly and salinity anomaly, used to reveal the hydrological structure of the eddy. (**a**) The eddy-composite analysis results for the KE1 region, (**b**) the eddy-composite analysis results for the KE2 region, (**c**) the eddy-composite analysis results for the STCC1 region, (**d**) the eddy-composite analysis results for the STCC2 region.

As shown in Figure 3, the temperature within the eddy core remains higher than the surrounding environment from the sea surface down to the subsurface layer. This phenomenon is a typical manifestation of downwelling within an anticyclonic eddy. In the Northern Hemisphere, anticyclonic eddies induce convergence in the Ekman layer, pumping warm surface water downward and suppressing the upwelling of colder underlying water. As a result, the entire water column within the eddy core is systematically warmer than the ambient environment, forming a warm-core structure. The eddy core also exhibits a high-salinity signature, though its vertical structure is less coherent than the temperature anomaly. The downwelling similarly transports high-salinity surface water (e.g., from the Kuroshio) into deeper layers. However, salinity distribution is more strongly influenced by water mass origins and mixing processes; therefore, the salinity anomaly is generally weaker and less uniform than the temperature anomaly. Four subplots present the eddy-composite results for four distinct regions, each depicting the characteristic structures of eddies in that area. Owing to differences in background environmental conditions and topography, the hydrographic distributions of eddies vary across these regions. Salinity is now defined as a ratio of electrical conductivities. It is no longer measured in parts per thousand but is expressed in practical salinity units (psu). It should be noted that the salinity values mentioned in this article are all expressed in units of psu.

### 3.2. Cyclone Eddy

**Figure 4:**
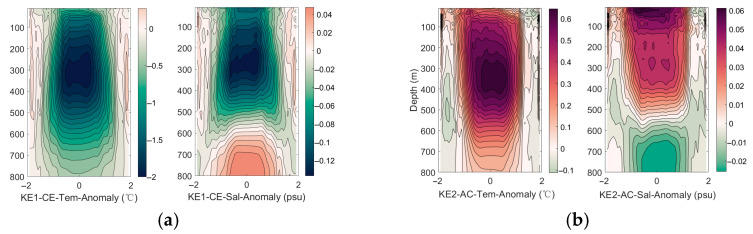

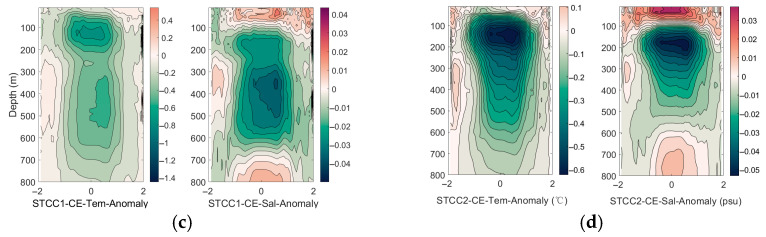
The horizontal axis represents the normalized radius, indicating the relative distance from the center of the cyclone eddy (normalizing the radius of the eddy to 1). The range of −2 to 2 indicates the distance extending approximately twice the radius of the eddy outward from the center of the eddy. The coloring variable is temperature anomaly and salinity anomaly, used to reveal the hydrological structure of the eddy. (**a**) the eddy-composite analysis results for the KE1 region, (**b**) the eddy-composite analysis results for the KE2 region, (**c**) the eddy-composite analysis results for the STCC1 region, (**d**) the eddy-composite analysis results for the STCC2 region.

Figure 4 reveals the two-dimensional distribution of the outliers. The tilt of the isopleths reveals the baroclinic nature of the eddy, meaning its structure varies with depth. Cyclonic eddies are typically characterized by a cold core, rotate counterclockwise in the Northern Hemisphere, and exhibit a sea surface depression. Their Ekman transport mechanism involves surface divergence, which induces upwelling. This upwelling brings cold, low-salinity, and nutrient-rich deep water to the upper ocean, forming a cold core and a low-salinity core. The temperature anomaly within the eddy core is lower than that in the peripheral region. The upwelling continuously transports cold deep water upward, resulting in temperatures within the eddy core—from the surface to the subsurface—being lower than those in the ambient ocean, thereby forming a vertically coherent cold core. The core exerts the strongest lifting and isolating effect on the internal water masses, while the outer region reflects more interaction between the eddy and the surrounding environment (e.g., Kuroshio water). This contrast indicates a strong radial gradient in the eddy’s influence. It can be observed that the cyclonic eddies and anticyclonic eddies within the STCC1 area all exhibit a multi-polar structure, and this structure is closely related to the specific geographical setting. The complex water mass distribution in region STCC1—such as the high-salinity Kuroshio water and low-salinity Oyashio water—is stirred by the eddy, leading to highly complex and asymmetric multipole structures, particularly in the salinity field. This distinguishes it clearly from eddies observed in other regions.

After obtaining the temperature and salinity anomalies, their anomaly values are added to the temperature and salinity data of the same background profile to derive the temperature and salinity fields. Based on the empirical sound speed formula, the relationship between sound speed, temperature, and salinity is defined as:

$$\begin{array}{c} c=1449.2+4.6T-0.055T^{2}+0.002T^{3}\\ +(1.34-0.010T)(S-35)+0.016D \end{array}$$

where c is the sound speed (m/s), T is the seawater temperature (°C), S is the seawater salinity (psu), and D is the depth (m). Based on the conversion from the temperature field and salinity field, the sound speed field under perturbation is obtained, and then subtracting the background sound speed yields the sound speed anomaly.

**Figure 5:**
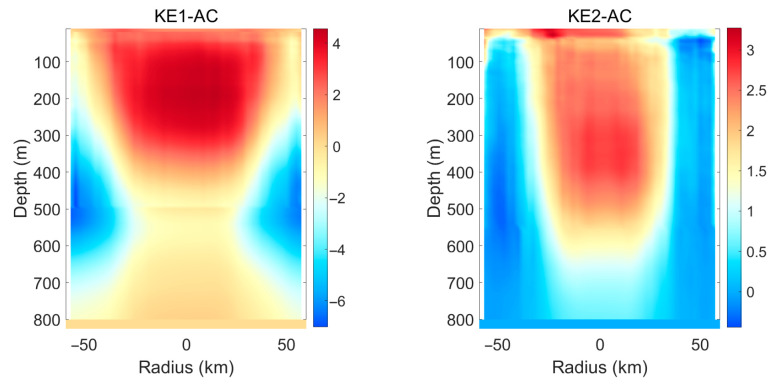

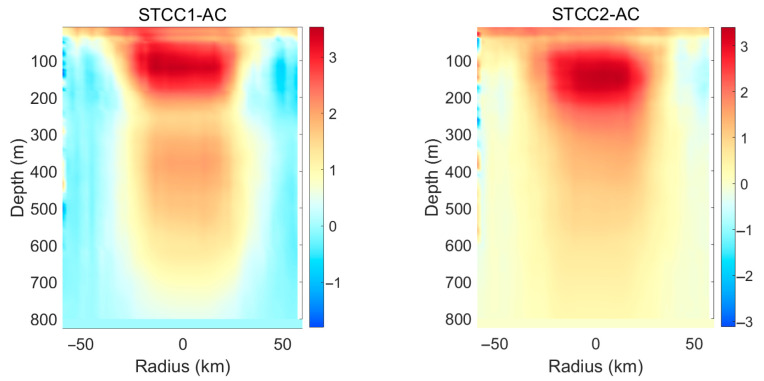
Anomaly of sound speed under the influence of the composite structure of anticyclonic eddies in different regions.

**Figure 6:**
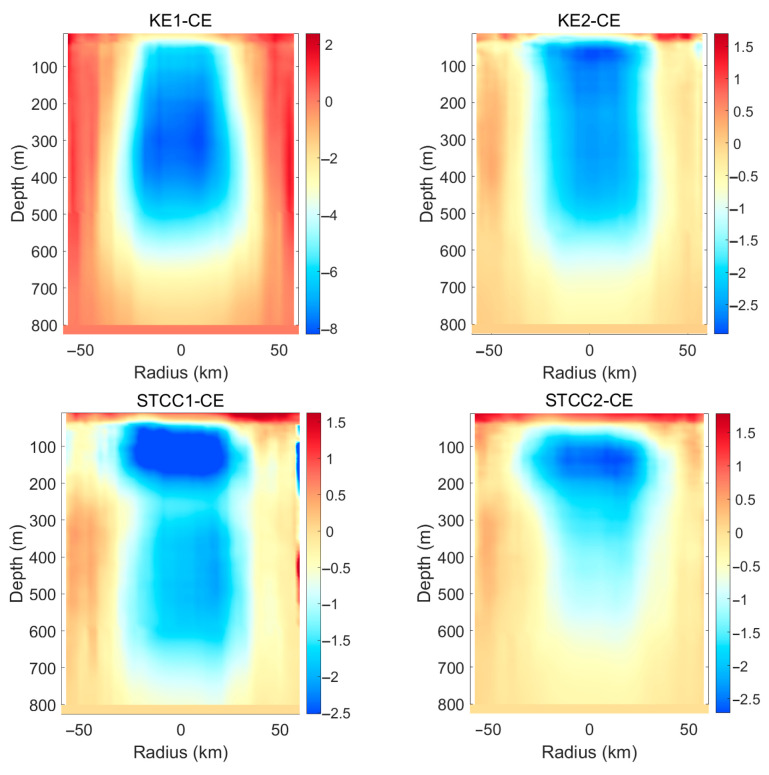
Anomaly of sound speed under the influence of the composite structure of cyclonic eddies in different regions.

As shown in Figure 5 and Figure 6, anticyclonic eddies are generally warm eddies; under their influence, sound speed increases, resulting in positive sound speed anomalies. Cyclonic eddies are generally warm eddies as well, but their sound speed decreases, leading to negative sound speed anomalies. In seawater, sound speed fields are primarily controlled by temperature fields: a 1 °C rise in temperature approximately increases sound speed by 2–4 m/s. A 1 psu increase in salinity roughly raises sound speed by 1 m/s. Salinity anomalies induced by eddies are often less than 0.05 psu, so sound speed variations are barely affected by salinity. Therefore, the morphology of sound speed anomalies closely resembles that of temperature anomalies.

### 3.3. Distance Estimation Error Under the Perturbation of the Gaussian Eddy Model

**Figure 7:**
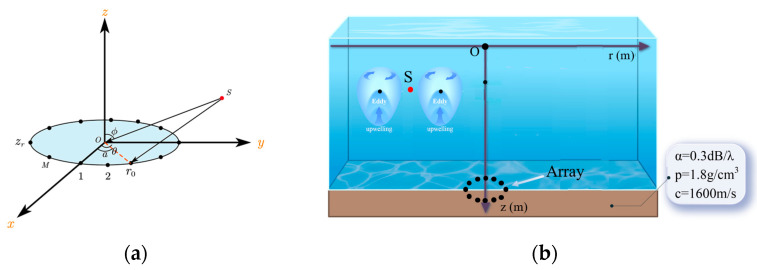
Schematic of the scenario. (**a**) Relative positions between the receiving array and the sound source. (**b**) Relative positions of the eddy, sound source, and receiving array. The coordinate relationship between (**a**) and (**b**) is: $r^{2}=x^{2}+y^{2}$. The coordinate system used in the simulation is shown in (**b**).

Figure 7 is a schematic diagram of the circular array. Consider a circular array consisting of 16 elements with an inter-element spacing of 5 m. The depth of this array, denoted as $z_{r}$, is 1245 m. S denotes the sound source. A cylindrical coordinate system is adopted, with the center of the circle as the origin and the *x*-axis passing through two array elements. The sound source S is located at an azimuth angle (α) of 30° relative to the *x*-axis, at a range of 7000 m from the center and a depth of 40 m. The source is broadband with a frequency range of 50–75 Hz. The water depth is 1245 m. The sound speed profile is shown in Figure 1. The seabed is modeled as a semi-infinite half-space with a P-wave sound speed of 1600 m/s, an attenuation coefficient of 0.3 dB/λ, and a density of 1.6 g/cm^3^. The signal-to-noise ratio (SNR) is set to 20 dB, where the noise is modeled as complex Gaussian white noise, and the SNR is defined relative to the clean signal power at the target position. To ensure the accuracy of the experiment, it is essential to verify that no localization error exists when the environmental and system parameters are properly matched. The search space is defined as a rectangular grid whose horizontal (range) axis extends from 5500 m to 8500 m with a 1 m step size, and whose depth axis spans 1 m to 200 m with a 0.5 m step size.

**Figure 8:**
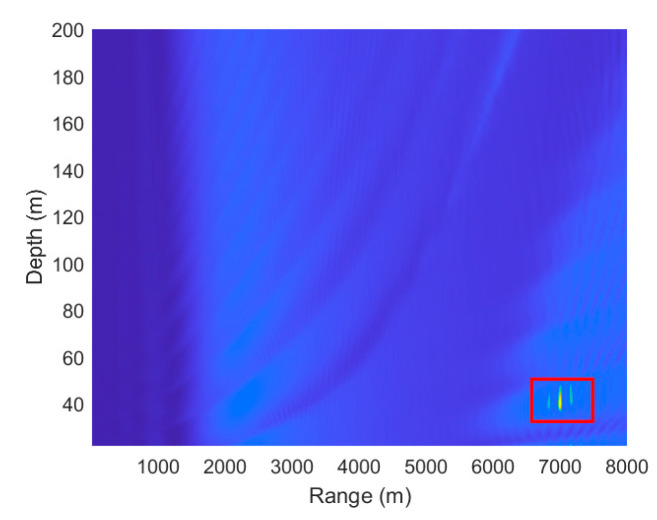
Target localization results under environmentally matched conditions.

**Figure 9:**
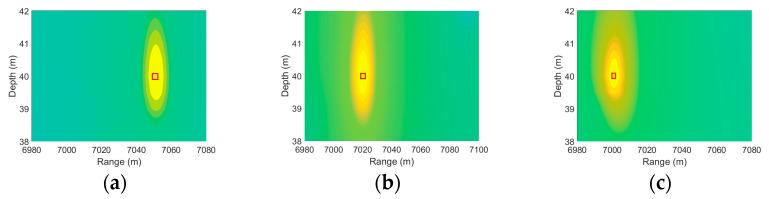
Ambiguity function peak shifting as a eddy moves away from the sound source at (7000 m, 40 m), with its center (**a**) 0 m, (**b**) 40 km, and (**c**) 80 km to the right.

**Figure 10:**
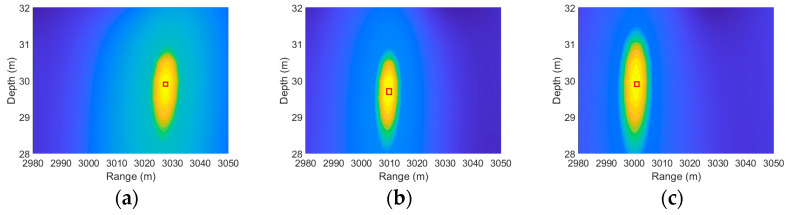
Ambiguity function peak shifting as a eddy moves away from the sound source at (3000 m, 30 m), with its center (**a**) 0 m, (**b**) 40 km, and (**c**) 80 km to the right.

As shown in Figure 8, the localization result is accurate under environmentally matched conditions and correct system parameters. The ambiguity surface in Figure 9 clearly shows that the range and depth coordinates corresponding to the global maximum peak align closely with the preset true sound source position (7000 m, 40 m). This demonstrates that the algorithm itself introduces no systematic errors under the current environmental model and parameter settings. Consequently, when disturbances such as mesoscale eddies are introduced, any resulting localization deviation can be unambiguously attributed to environmental mismatch rather than defects in the algorithm. This establishes a solid foundation for quantitatively analyzing the impact of environmental uncertainty on localization performance. As shown in Figure 9 and Figure 10, as the eddy passes through the sound source, it causes a continuous shift in the peak of the ambiguity function. When the eddy center is 0 m away from the sound source, the eddy exerts the strongest influence, resulting in the maximum shift and the most severe positioning error. As the eddy gradually moves away from the sound source, its influence weakens, leading to a gradual reduction in both the peak shift and the range estimation error.

**Figure 11:**
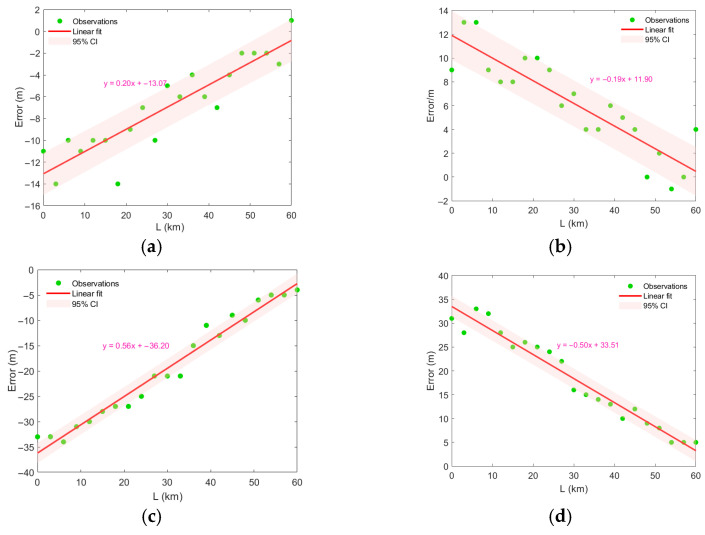

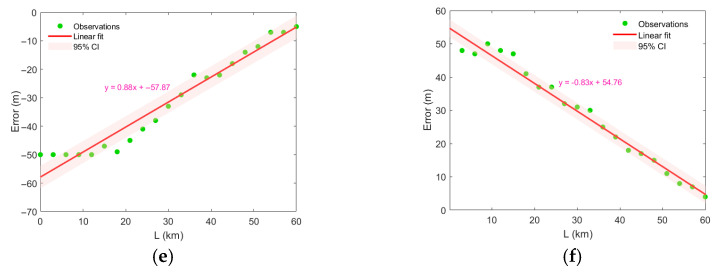
Variation in distance estimation error during eddy movement. (**a**) warm eddy, A = 2, (**b**) warm eddy, A = 2, (**c**) cold eddy, A = −6, (**d**) warm eddy, A = 6, (**e**) cold eddy, A = −10, (**f**) warm eddy, A = 10.

Using a Gaussian eddy model, it is assumed that initially the center of the Gaussian eddy is located directly below the sound source. As the eddy gradually traverses across the sound source, it causes continuous changes in the sound speed profile of the measured field, leading to mismatch in the sound speed profile. This Gaussian eddy has a radius of 40 km, an influence depth of 800 m, and a center depth of 300 m, with intensity values set to 2, 6, and 10 (negative values for cold-core eddies). Under these conditions, the localization error induced by the eddy exhibits a regular variation. It should be noted that the term localization error in this paper specifically refers to range estimation error, and does not include errors in depth estimation or bearing estimation. No significant correlation was observed between depth estimation error and the degree of mismatch in the experiments. Therefore, depth error was not analyzed or discussed. As shown in Figure 11, cold-core eddies cause an underestimation of range, while warm-core eddies lead to an overestimation. Meanwhile, the localization error is closely related to the influence of the eddy on the sound speed at the source position. As the eddy moves away from the source, the degree of mismatch in the sound speed profile continuously decreases, and the range estimation error also progressively reduces.

Fitting the relationship between the eddy’s travel distance and the localization error reveals an approximately linear trend, whose slope depends on the type and intensity of the eddy. Cold-core eddies exhibit a positive slope, while warm-core eddies show a negative slope. The term “95% Confidence Interval (95% CI)” refers to a statistical range within which there is 95% certainty that the true value of a population parameter lies. A high goodness-of-fit is often accompanied by a narrow confidence interval. The narrower the confidence interval, the more reliable the fitted model is considered, as it reflects greater precision in parameter estimation and stronger predictive consistency. The greater the eddy intensity, the larger the absolute value of the slope. It is observed that stronger eddy intensities result in narrower confidence interval bands (shaded areas), indicating a stronger correlation.

**Figure 12:**
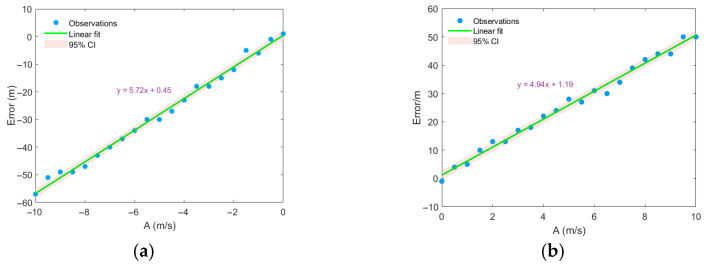
Distance estimation error varies with eddy intensity. (**a**) cold eddy, (**b**) warm eddy.

To investigate the correlation between range estimation error and eddy intensity, the eddy was assumed to be stationary with its center directly below the sound source. The eddy center was set at a depth of 300 m, with a radius of 40 km and an influence depth of 800 m. By varying the intensity of the Gaussian eddy, a strong correlation between eddy intensity and range estimation error was observed. As shown in Figure 12, the smaller the eddy intensity (the absolute value of A), the smaller the range estimation error. This occurs because a weaker eddy introduces less mismatch in the sound speed profile.

**Figure 13:**
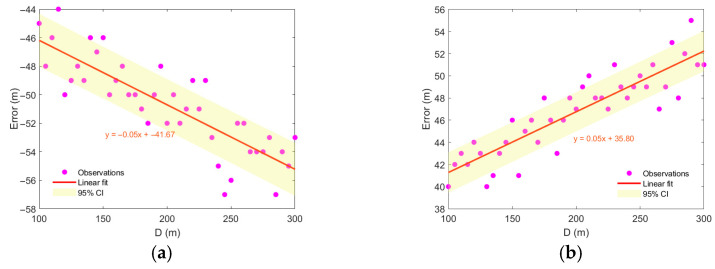
Range estimation error varies with the depth D of the Gaussian eddy center. (**a**) cold eddy, (**b**) warm eddy.

The eddy was assumed to remain stationary, with its center aligned with the sound source along the *z*-axis. The radius was set to 40 km, the influence depth to 800 m, and the eddy intensity to 10. While keeping all other parameters in Equation (2) unchanged, modifying the depth D of the Gaussian eddy center results in statistically regular variations in localization error. As shown in Figure 13, a strong correlation exists between the variation in the central depth of the Gaussian eddy and the resulting localization error. A nearly linear relationship was observed between the range estimation error and the central depth of the eddy. Cold-core eddies exhibit a negative slope, whereas warm-core eddies show a positive slope. This behavior occurs because, in the Gaussian eddy model, changing only the central depth of the eddy—while keeping other parameters constant—modifies the sound speed profile around the sound source to a certain extent.

**Figure 14:**
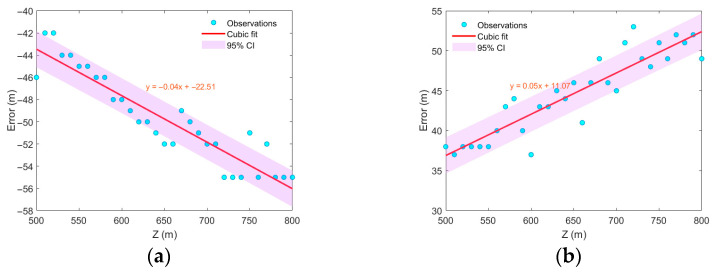
Range estimation error varies with the depth Z of Gaussian eddy influence. (**a**) cold eddy, (**b**) warm eddy.

Similarly, varying the influence depth of the Gaussian eddy also leads to corresponding changes in localization error. While keeping all other parameters in Equation (2) unchanged, modifying the influence depth Z of the Gaussian eddy results in statistically regular variations in localization error. The underlying cause of this behavior is the same as previously explained. However, the correlation between the influence depth of the eddy and the resulting localization error is relatively weak—significantly weaker than the correlation observed with eddy intensity. As shown in Figure 14, a fitted relationship between these two variables yields a slope with a small absolute value.

### 3.4. Impact of the Synthetic Vertical Structure of Mesoscale Eddies in Different Regions of the Northwestern Pacific on Localization Error

Precise underwater localization is a critical requirement for marine scientific research, national defense security, and resource exploration. Although Matched Field Processing (MFP) technology has the potential for high precision, its performance heavily depends on the accuracy of the ocean sound speed field. Mesoscale eddies, as ubiquitous dynamic phenomena in the ocean, are one of the key factors causing spatiotemporal variability in the sound speed field. Previous studies have primarily focused on the impact of eddies on acoustic transmission loss or provided case-specific statistics of localization errors, lacking a systematic investigation into the quantitative mechanisms through which eddies with different characteristics (from various oceanic regions and possessing distinct three-dimensional structures) affect MFP localization errors. This work synthesizes the typical vertical thermohaline structures of mesoscale eddies from four key regions in the Northwestern Pacific. Using these structures as input, it quantitatively simulates and analyzes their influence on the accuracy distribution of MFP localization, revealing the intrinsic relationship between the magnitude of errors and the physical features of the eddies.

Assuming an eddy radius of 10 km, the three-dimensional structures illustrated in Figure 4 and Figure 6 are scaled proportionally to a spatial extent of 40 km. Since the background profile provides only the sound speed field, and the relationship between sound speed and temperature, salinity, and density is not a simple linear superposition, it is necessary to invert the temperature field from the background sound speed profile using an empirical sound speed equation. In practice, given that salinity variations and their impacts are negligible, salinity can be held constant at 35 psu.The Newton iteration method is then applied to retrieve the temperature field corresponding to the background sound speed profile. The eddy-induced perturbations are superimposed onto this background temperature and salinity field, and finally, the sound speed field under eddy influence is computed using the empirical sound speed formula [38].

Idealized eddy models often overlook the complexity of the marine environment. In contrast, using synthesized vertical structures of regional mesoscale eddies allows for the retention of environmental complexity while enabling the investigation of the overall structural characteristics of eddies in a given region. By analyzing the impact of synthesized mesoscale eddy structures from different regions on matched-field localization error, we can explore the statistically significant effects of mesoscale eddies on localization across various areas.

**Figure 15:**
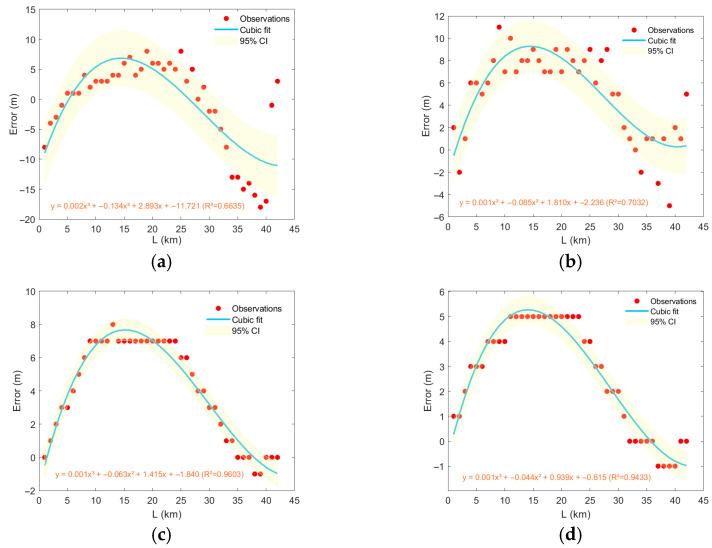
Estimation errors in the distance caused by the propagation of anticyclonic eddies in different regions. (**a**) region KE1, (**b**) region KE2, (**c**) region STCC1, (**d**) region STCC2.

**Figure 16:**
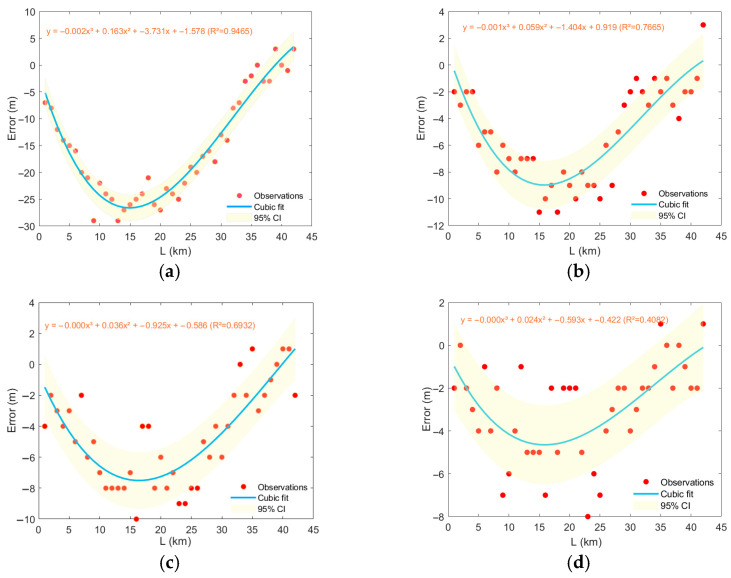
Estimation errors in the distance caused by the propagation of cyclonic eddies in different regions. (**a**) region KE1, (**b**) region KE2, (**c**) region STCC1, (**d**) region STCC2.

This study investigates the influence of mesoscale eddies on Conventional Matched Field Processing performance through four sets of numerical experiments. Figure 15 and Figure 16 demonstrate the variation in localization error with the movement of typical mesoscale eddies from different regions: the error exhibits significant nonlinear behavior with increasing distance—initially increasing rapidly, reaching a peak in the intermediate range, and then gradually decaying. A cubic polynomial fit indicates that this relationship is highly interpretable.

Errors in range estimation caused by anticyclonic eddies follow a parabolic trend with travel distance, first positive and then negative. Eddies from different regions (e.g., the Kuroshio Extension vs. the Oyashio-influenced area) show distinct differences in sound propagation perturbation and final localization error due to variations in their thermohaline structure, intensity, and vertical extent. Regarding localization error induced by anticyclonic eddies, regions KE1 and KE2 exhibit stronger impacts than region STCC1, with region STCC2 being the weakest. For cyclonic eddies, region KE1 shows the strongest impact, followed by regions KE2 and STCC1, with region STCC2 again being the weakest. As a mesoscale eddy gradually intrudes into the vicinity of the sound source, the localization error increases rapidly and converges toward a peak. This phase corresponds to the core region of the eddy, where enhanced hydrographic perturbation leads to increased localization deviation. This trend is roughly the same in different regions, but the extreme values reached are different.

## 4. Discussion

In beamforming or matched field processing, localization relies on the phase relationships of the acoustic pressure signals received by the array. The phase slope is defined as the rate of change in phase with respect to the sensor index, representing the phase variation across each array element. Taking linear array beamforming as a more intuitive example, the phase slope reflects the variation in the arrival angle of the acoustic wave at the array. If the phase slope is constant, it indicates that the acoustic wave arrives at the array at a fixed angle, causing the beamformer to steer toward that direction. If the angle between the source direction and the array normal is θ,the phase difference between adjacent array elements is given by:

$$\Delta \phi =\frac{2\pi d}{\lambda }\sin\theta$$

where d is the element spacing and λ is the wavelength. When sound speed profile mismatch occurs, the actual sound speed differs from the model sound speed, leading to bending of the acoustic propagation path and thus altering the phase of the signal arriving at the array. The change in phase slope directly modifies the angle estimated by the beamformer, resulting in localization error. In other words, the localization error is correlated with variations in the phase slope. For a linear array:

$$\varepsilon_{\theta }\propto \Delta s$$

where Δs denotes the phase slope and $\varepsilon_{\theta }$ represents the angular estimation error. In matched field processing, the underlying principle is analogous but more complex due to the involvement of the entire propagation environment. Essentially, however, variations in the phase slope cause phase mismatch between the replica field and the measured field, leading to a shift in the peak of the ambiguity surface and resulting in localization error. Although the phase variation in a circular array is not governed by a simple linear relationship, analyzing its fitted slope remains valuable for understanding the distribution of localization errors.

**Figure 17:**
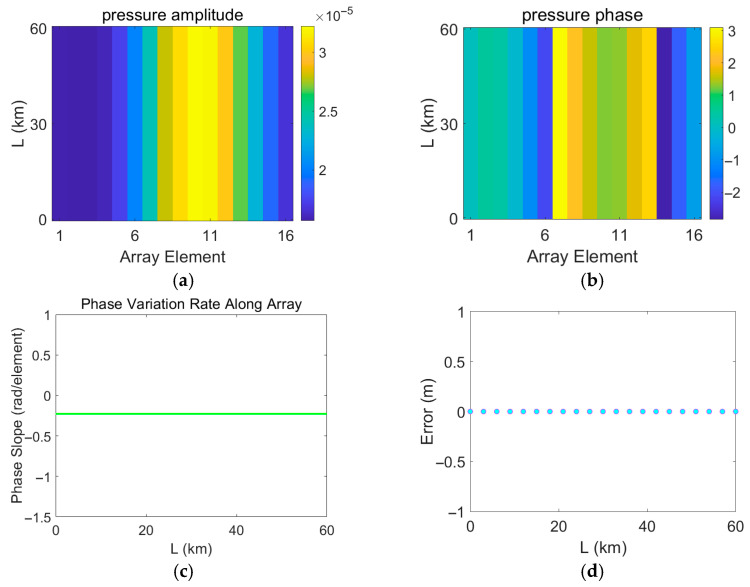
Distribution of errors induced by eddy movement and variations in the measured pressure field under eddy intensity A = 0. (**a**) Amplitude of the measured pressure field; (**b**) Phase of the measured pressure field; (**c**) Range estimation error; (**d**) Phase slope. (**a**,**b**), the horizontal axis represents the propagation distance of the eddy L, and the vertical axis corresponds to the sensor index. Values are color-mapped to represent the amplitude and phase, respectively.

**Figure 18:**
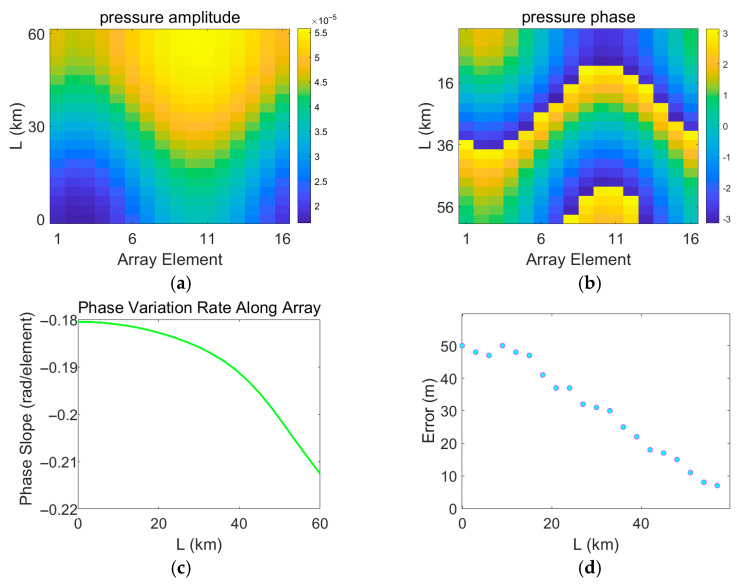
Distribution of errors induced by eddy movement and variations in the measured pressure field under eddy intensity A = 10. (**a**) Amplitude of the measured pressure field; (**b**) Phase of the measured pressure field; (**c**) Range estimation error; (**d**) Phase slope. (**a**,**b**), the horizontal axis represents the propagation distance of the eddy L, and the vertical axis corresponds to the sensor index. Values are color-mapped to represent the amplitude and phase, respectively.

**Figure 19:**
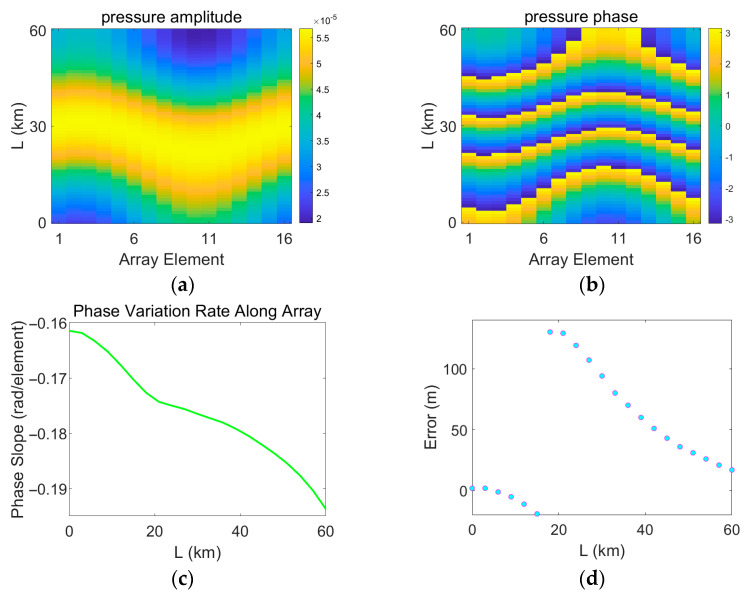
Distribution of errors induced by eddy movement and variations in the measured pressure field under eddy intensity A = 30. (**a**) Amplitude of the measured pressure field; (**b**) Phase of the measured pressure field; (**c**) Range estimation error; (**d**) Phase slope. (**a**,**b**), the horizontal axis represents the propagation distance of the eddy L, and the vertical axis corresponds to the sensor index. Values are color-mapped to represent the amplitude and phase, respectively.

**Figure 20:**
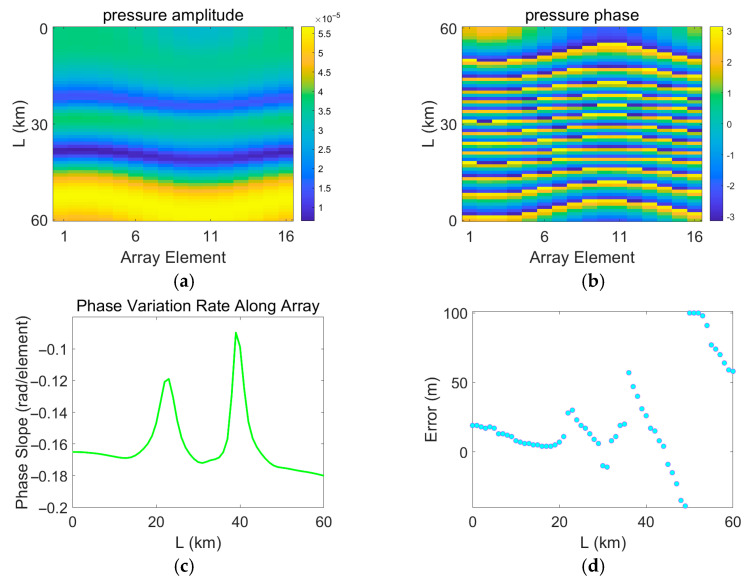
Distribution of errors induced by eddy movement and variations in the measured pressure field under eddy intensity A = 100. (**a**) Amplitude of the measured pressure field; (**b**) Phase of the measured pressure field; (**c**) Range estimation error; (**d**) Phase slope. (**a**,**b**) the horizontal axis represents the propagation distance of the eddy L, and the vertical axis corresponds to the sensor index. Values are color-mapped to represent the amplitude and phase, respectively.

In the process of error measurement, it is assumed that the measured field is continuously disturbed by a mesoscale eddy, while the replica field is calculated based on a sound speed profile measured in the absence of an eddy. Therefore, the measured field continuously changes, whereas the replica field remains unchanged. The variations in the acoustic pressure of the measured field are shown in Figure 17, Figure 18, Figure 19 and Figure 20. Figure 17, Figure 18, Figure 19 and Figure 20 depict the amplitude and phase variations in the acoustic pressure in the measured field as the Gaussian eddy moves. And itrepresents the slope obtained by performing linear fitting on the unwrapped phase values taken from all sensor elements at that specific range. The phase slope reflects the variation in the degree of mismatch.

As shown in Figure 17, when the eddy strength is set to 0 (equivalent to no eddy), running the program yields simulation results. In this case, eddy movement does not induce range estimation errors. Additionally, the sound pressure does not vary with the eddy movement distance but only with the array element sequence. This validates the correctness of the program and confirms that the positioning errors in the simulation results do not originate from the program itself.

As shown in Figure 18, when the eddy intensity is low and the sound speed mismatch is minimal, the localization error increases linearly with the degree of mismatch. The phase of the measured field changes continuously and smoothly, corresponding to a continuous and smooth deviation growth between the replica field phase and the measured field phase as the eddy moves. However, sound speed mismatch does not always induce predictable changes: in many cases, the relationship between the degree of mismatch and the localization error is highly random.

As shown in Figure 19 and Figure 20, when the eddy intensity is set to 30 or 100, the relationship between the degree of mismatch and the localization error clearly deviates from the linear pattern described above. Of course, such intense eddies do not exist in the actual ocean. An eddy intensity of 10 already represents an extreme scenario, implying that the water temperature at the eddy core is about 2 °C higher than that of the surrounding water. To examine scenarios where the sound speed mismatch exceeds a certain threshold and leads to nonlinear behavior in localization error, the eddy intensity was artificially set to unrealistic values of 30 and 100. In Figure 19, when the eddy intensity is high and the degree of mismatch increases beyond a certain point, the localization error jumps abruptly after reaching a critical value. This corresponds to a situation where the main correlation peak is surpassed by a sidelobe, indicating a mode jump of the maximum peak. In Figure 20, when the mismatch is very severe, the localization error becomes irregular and constantly jumps. Furthermore, comparing Figure 20c,d, it can be observed that the locations where the error and the phase slope change abruptly are generally consistent. This demonstrates the strong correlation between the error distribution and the phase variation. As mentioned earlier, the regularity of the error distribution is ultimately attributable to uniform and continuous changes in the sound speed profile, which lead to smooth and continuous deviations between the phases of the measured and replica fields. The causes of these jumps are complex. They may result from fundamental alterations in the phase structure of the sound field due to severe sound speed mismatch, or from interference-induced phase strengthening and cancelation effects related to multipath propagation.

## 5. Conclusions

In summary, under idealized conditions that exclude small-scale processes such as turbulence and internal waves, mesoscale eddies—as large-scale, weak-disturbance oceanic dynamical phenomena—induce limited and predictable systematic errors in underwater acoustic target localization. Moreover, key eddy parameters (e.g., intensity, core depth, and relative position) exhibit significant correlations with the magnitude and distribution of localization errors. This finding provides an important theoretical basis and a novel approach for inverting oceanographic parameters of mesoscale eddies using acoustic observation methods such as matched-field localization (MFP). This implies that underwater acoustic sensor networks, while fulfilling their primary mission of localization, could also serve as distributed sensing systems for remotely monitoring large-scale oceanic dynamical processes that are otherwise difficult to measure directly.

However, applying this theory to real-world marine environments poses considerable challenges. Idealized, pure mesoscale eddies do not exist in nature; they are inherently embedded within a complex multi-scale dynamical background as “hybrid” structures. Their interiors and peripheries are often accompanied by submesoscale eddies, fronts, and filaments generated by shear instability. These finer-scale processes introduce high-frequency, stochastic phase and amplitude fluctuations. Furthermore, ubiquitous internal waves and turbulence cause scattering and signal fluctuations in the acoustic field. Therefore, bridging the significant gap from strong theoretical correlation to reliable engineering application requires overcoming the complexities of the marine environment. The key to achieving this transition lies in developing advanced processing algorithms and observational strategies capable of effectively extracting weak eddy signals from multi-scale, high-noise background fields.

## Figures and Tables

**Table 1:** The four sub-regions defined for the statistical analysis of mesoscale eddies and their longitudinal and latitudinal extents.

Region	Longitude/Latitude
KE 1	135° E–165° E, 30° N–45° N
KE 2	165° E–195° E, 30° N–45° N
STCC1	135° E–165° E, 15° N–30° N
STCC2	165° E–195° E, 15° N–30° N

## Data Availability

The calculation of the sound field relies on the software KRAKEN (Version 2020) in the Acoustic Toolbox, which is available at http://oalib.hlsresearch.com/AcousticsToolbox/ (accessed on 12 August 2023). The Acoustics Toolbox is a specialized computational toolkit developed by Michael B. Porter and collaborators for modeling underwater acoustic propagation in marine environments. It integrates numerical methods such as ray tracing, normal mode theory and parabolic equation approaches, offering models like KRAKEN, Bellhop and RAM to simulate acoustic fields under diverse oceanic conditions. The Acoustics Toolbox is distributed under the GNU Public License.
